# Multiplex RT-qPCR Application in Early Detection of Bovine Respiratory Disease in Healthy Calves

**DOI:** 10.3390/v15030669

**Published:** 2023-03-02

**Authors:** Yusuke Goto, Kazuhiro Fukunari, Tohru Suzuki

**Affiliations:** 1 Central Iwate Prefectural Livestock Health and Hygiene Center, Takizawa 020-0605, Iwate, Japan; 2 Division of Zoonosis Research and Division of Hygiene Management, Sapporo Research Station, National Institute of Animal Health, NARO, Sapporo 062-0045, Hokkaido, Japan

**Keywords:** bovine respiratory disease, multiplex real-time RT-PCR, calf, early detection, monitoring, virus neutralization test, viral pathogens, bacterial pathogens

## Abstract

Bovine respiratory diseases (BRD) are associated with various predisposing factors, such as physical and physiological stress factors, and bacterial and viral pathogens. These stressors and viruses suppress immune defenses, leading to bacterial growth in the upper respiratory tract and invasion of pathogens into the lower respiratory tract. Therefore, continuous monitoring of the causative pathogens would contribute to the early detection of BRD. Nasal swabs and sera from 63 clinically healthy calves were continuously collected from seven farms in Iwate prefecture from 2019 to 2021. We attempted to monitor dynamics of BRD-associated pathogens by multiplex real-time RT-PCR (RT-qPCR) using their nasal swab samples. In addition, we attempted to monitor fluctuation of antibody titers against each BRD-associated pathogen by virus neutralization test (VNT) using their sera. In contrast, nasal swabs from 89 calves infected with BRD were collected from 28 farms in Iwate prefecture from 2019 to 2021. We attempted to analyze their nasal swab samples by multiplex RT-qPCR aim to detect BRD-associated pathogens that are dominant in this region. As a result, our analyses using samples from clinically healthy calves showed that positive results by multiplex RT-qPCR were closely related to a significant increase of antibody titers by VNT in bovine coronavirus (BCoV), bovine torovirus (BToV), and bovine respiratory syncytial virus (BRSV). In addition, our data exhibited that BCoV, BToV, BRSV, bovine parainfluenza virus 3, and *Mycoplasma bovis* have been more frequently detected in calves infected with BRD compared to those detected in clinically healthy calves. Moreover, the data presented herein revealed co-infections by combination multiple viral pathogens with bacterial pathogens are closely involved in the onset of BRD. Taken together, our study demonstrates multiplex RT-qPCR which can simultaneously analyze multiple pathogens, including viruses and bacteria, and is useful for the early detection of BRD.

## 1. Introduction

Bovine respiratory disease (BRD) causes globally severe economic losses due to increased rates of morbidity, mortality, treatment costs, and loss of production [1,2,3]. BRD is associated with various predisposing factors, such as physical and physiological stress factors, multiple pathogens (viruses and bacteria), health condition of host, and environ-mental factors. Of them, bacterial and viral pathogens are important factors of BRD onset [4,5,6]. The major BRD-associated viral pathogens include pestiviruses A and B (known as bovine viral diarrhea virus (BVDV) 1 and 2), bovine herpes virus 1 (BHV1), bovine parainfluenza virus 3 (BPIV3), bovine respiratory syncytial virus (BRSV), bovine coronavirus (BCoV), and bovine adenovirus (BAdV) [7]. In addition, bovine influenza D virus (BIDV) and bovine torovirus (BToV) are also considered to be BRD-associated pathogens [8,9,10]. In contrast, the major BRD-associated bacterial pathogens include *Mannheimia haemolytica* (Mh), *Pasteurella multocida* (Pm), *Mycoplasma bovis* (Mb), and *Histophilus somni* (Hs) [11].

In general, several potentially BRD-associated bacterial pathogens are asymptomatic and present in the nasal cavity of cattle, of which the most common pathogens are Mh and Pm [12]. In healthy cattle, a delicate balance exists between these potentially bacterial pathogens and the indigenous microflora of the upper respiratory tract, and moreover, the primary immune mechanism actively prevents the establishment of pathogens into the lower respiratory tract. However, BRD occurs because this homeostatic balance in the upper respiratory tract is disrupted, and pathogens invade the lower respiratory tract [13].

To detect BRD onset early in cattle herd, it is important to continuously monitor the dynamics of major causative pathogens [14]. There are many previous reports concerning the involvement of various pathogens in BRD by testing with PCR and real-time PCR using nasal swabs from infected cattle [15,16]. Furthermore, various investigations based on comparison between nasal swabs from healthy and BRD-infected cattle have been conducted to elucidate a mechanism of BRD onset by pathogens [17,18,19]. To our knowledge, however, there are few reports that monitor the causative pathogens, a sign of BRD onset in healthy cattle for a long period.

The detection of genes from pathogen by a PCR is rapid and useful, suggesting the association of specific pathogens with the disease [20,21]. However, further tests are needed, including measuring antibody titer and monitoring the dynamics of virus genes, to confirm whether an animal is actually infected with the pathogen [22,23]. In addition, we need to fully understand the existence of pathogens in the nasal cavity of healthy cattle [24,25,26].

In this study, we attempted to detect BRD onset early in herds by analyzing the presence of BRD-associated pathogens in nasal swabs and their relationship to BRD onset in healthy cattle with a multiplex real-time RT-PCR (RT-qPCR). The data presented in this study will provide useful information around early detection and mechanism elucidation of BRD onset.

## 2. Materials and Methods

### 2.1. Sampling from Clinically Healthy Calves

Samples from 63 calves maintained in seven farms (4 dairies and 3 nurseries) in Iwate prefecture from 2019 to 2021 are summarized in Supplementary Table S1. Three calves with good health condition per farm were selected for the first time every year and were followed continuously for one year, and then they were changed each year. Nasal swabs and sera were collected four times (June, August, September, and November) per year. Finally, a total of 232 nasal swabs and sera were collected from 20 sample collections. The health conditions of the selected calves during the investigation period were interviewed with each farmer. If those calves had a symptom during the period, they were diagnosed by veterinarians with experiences and knowledge.

### 2.2. Sampling from Calves with Respiratory Symptoms

Nasal swabs were collected from 89 calves with respiratory symptoms, aged 5 days to 12 months, from 28 farms in Iwate prefecture during 2019 to 2021. The symptoms of all calves were diagnosed by veterinarians with experience and knowledge. One calf originated from the farm G, and the remaining 88 calves were derived from 27 farms other than the above seven farms (Supplementary Table S1). Of 89 calves, 23 calves were vaccinated against 5 viruses (BVDV, BAdV7, BRSV, BPIV3, and BHV1), and 9 calves were vaccinated against 2 viruses (BPIV3 and BHV1), respectively.

### 2.3. Extraction of DNA and RNA from Pathogens

Nasal swabs collected by a cotton stick were suspended in 2 mL PBS and mixed gently. DNAs and RNAs of pathogens were extracted from the suspensions using a QIAamp MinElute Virus Spin kit (QIAGEN, Hilden, Germany) according to the manufacturer’s instruction. In addition, the nucleic acids were also extracted from RNase-free water, as were the samples, and were used as a negative control.

### 2.4. Multiplex Real-Time RT-PCR (Multiplex RT-qPCR) Assay

TaqPath 1-step Multiplex Master Mix (Thermo Fisher Scientific, Carlsbad, CA, USA) was used as a reagent of multiplex real-time RT-PCR (RT-qPCR). The used primers and probes are described in our previous study (Supplementary Table S2) [27], which were purchased from Thermo Fisher Scientific (Thermo Fisher Scientific, Carlsbad, CA, USA). Twelve pathogens were divided into three units consisting of four pathogens; Unit 1, BVDV, BCoV, BToV, and BAdV; Unit 2, BRSV, BPIV3, BIDV and BHV1; Unit 3, Mb, Mh, Pm, and Hs.

The reaction volume per one unit was 20 μL, including 5 μL TaqPath 1-step Multiplex Master Mix, 1 μL (total 4 μL) of mixture of forward and reverse primers (900 nmol/L each) and probe (250 nmol/L) per pathogen, 4 μL of sample, and 7 μL RNase-free water. Multiplex RT-qPCR was conducted using an Applied Biosystems QuantStudio 5 Real-Time PCR System (Thermo Fisher Scientific, Carlsbad, CA, USA) under the following conditions: 25 °C for 2 min, 53 °C for 10 min, and 95 °C for 2 min, followed by 40 cycles of 95 °C for 3 s and 60 °C for 30 s. All results were analyzed using the QuantStudio Design and Analysis Software (Version 1.5).

In the analysis by multiplex RT-qPCR, the cut-off value for pathogen detection was set at Ct 40 or less (Ct value ≤ 40). In addition, the multiplex RT-qPCR analysis was performed in duplicate to robustly evaluate the possibility of non-specific reaction of the sample with high Ct values. The average of the two Ct values was used as the results. In addition, a retest was performed if no Ct value was obtained on one side, and cases when no Ct value was obtained on one side in the retest were considered to be negative.

### 2.5. Virus Neutralization Test (VNT)

The virus neutralization test (VNT) was performed according to previously reported methods [28,29]. Each 50 µL of serum was serially diluted two-fold with 50 µL EMEM supplemented with 3 mg/mL tryptose phosphate broth (Becton Dickinson, Sanjose, CA, USA), 0.292 mg/mL L-glutamine (FUJIFILM Wako Pure Chemical Corporation, Osaka, Japan), and 1.125 mg/mL sodium hydrogen carbonate (FUJIFILM Wako Pure Chemical Corporation, Osaka, Japan) on 96-well plates.

For Nose strain (BVDV1), KZ-91-CP strain (BVDV2), Los Angeles strain (BHV1) and Fukuroi strain (BAdV7), 50 µL (200 TCID_50_/0.1 mL per each virus) of these strains was added to well and incubated at 37 °C for 1 h. Thereafter, 100 µL of bovine fetal muscular cells (approximately 1.5 × 10^4^ cells) originally produced in our laboratory was added into all wells and incubated at 37 °C in 5% CO_2_ for 7 days.

For Kakegawa strain (BCoV) and Aichi strain (BToV), 50 µL (200 TCID_50_/0.1 mL per each virus) of these strains was added to well and incubated at 37 °C for 1 h. Thereafter, 100 µL of HRT-18G cells (approximately 1.5 × 10^4^ cells) was added into all of the wells and incubated at 37 °C in 5% CO_2_ for 7 days.

For 52-163-13 strain (BRSV) and BN-1 strain (BPIV3), 50 µL (200 TCID_50_/0.1 mL per each virus) of these strains was added to well and incubated at 37 °C for 1 h. Thereafter, 100 µL of Vero cells (approximately 1.5 × 10^4^ cells) was added into all wells and incubated at 37 °C in 5% CO_2_ for 7 days.

The appearance of the cytopathogenic effect (CPE) was observed using a microscope (Olympus Corporation, Tokyo, Japan). The VN titer for each serum sample was expressed as the reciprocal of the highest dilution that inhibited CPE.

**Figure 1:**
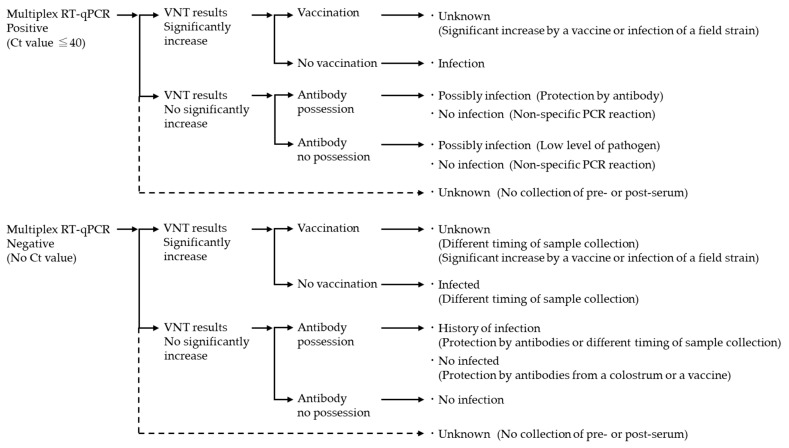
A flow chart to evaluate infection status based on results by multiplex RT-qPCR and results by VNT. “No collection of pre- or post-serum” means that the sample was not collected in a session before or after the session in which the result by multiplex RT-qPCR was positive.

An antibody titer of less than 2-fold was considered to be negative. A significant increase was defined as a 4-fold or greater increase in an antibody titer compared to two consecutive titers. For calves treated with vaccines containing BVDVs, BAdV7, BRSV, BPIV3, and BHV1, positive results obtained in each pathogen with multiplex RT-qPCR using nasal swabs from the animals, including cohabiting calves, were considered as a significant increase by the field strain or vaccine; otherwise, it was considered to be an effect of the vaccine. Furthermore, because cross-reactivity was observed between BVDV1 and BVDV2 antibodies, the significant increase of BVDV2 antibody titer in BVDV1 vaccinated calves was considered to be due to the vaccine [30,31]. A flowchart combined results by multiplex RT-qPCR with results of antibody titers by VNT is summarized in Figure 1.

### 2.6. Ethics Statement

Clinical samples were collected as a part of routine diagnostic procedures, including monitoring of other pathogens; hence, permission regarding animal ethics was not required.

## 3. Results

### 3.1. Confirmation of Respiratory Disease Onset in Clinically Healthy Calves

Throughout interviews with farmers and diagnosis by veterinarians with experiences and knowledge during the investigation period, we confirmed an onset of respiratory disease in seven calves (nos. B1–B3, D4–D6, and G6) and the death of two calves (nos. B1 and G6) from three farms (B, D, and G) (Supplementary Table S1). In addition, there were epidemic cases of respiratory disease on three farms (nos. B, D, and F) during the same period, and nine calves (nos. B7–B9, D7–D9, and F4–F6) were suspected to have the disease.

### 3.2. Detection of Pathogens Using 232 Nasal Swab Samples from 63 Clinically Healthy Calves

A total of 319 PCR-specific genes from nine pathogens were detected using 232 nasal swab samples from 63 clinically healthy calves collected in Iwate prefecture from 2019 to 2021. The pathogen-specific genes detected by multiplex RT-qPCR were Pm (number of positives 150/total number 232, percentage of positives 64.7%), Mh (73/232, 31.5%), Hs (33/232, 14.2%), BCoV (30/232, 12.9%), Mb (12/232, 5.2%), BAdVs (9/232, 3.9%), BRSV (5/232, 2.2%), BToV (5/232, 2.2%), and BPIV3 (2/232, 0.9%). Specific genes were not detected in BVDV 1 and 2, BIDV, and BHV1 by multiplex RT-qPCR (Table 1).

### 3.3. Virus Neutralization Test Using 232 Sera from 63 Clinically Healthy Calves

The antibody titers measured by VNT against each pathogen, using 232 sera from 63 clinically healthy calves, are summarized in Table 2. A significant increase was defined as cases that showed a 4-fold or greater increase than the antibody titer measured in the previous test. Based on our criteria, the samples that showed significant increases in antibody titers against each pathogen were BVDV1 (28), BVDV2 (24), BCoV (28), BToV (29), BAdV7 (38), BRSV (21), BPIV3 (38), and BHV1 (7). Of them, the number of vaccinated samples that could have influenced a significant increase of antibody titers against each pathogen were as follows: BVDV1 (28/28), BVDV2 (24/24), BCoV (0/28), BToV (0/29), BAdV7 (26/38), BRSV (12/21), BPIV3 (32/38), and BHV1 (7/7). Briefly, most samples with a significant increase in antibody titers were due to vaccines against each pathogen, except for BCoV and BToV.

### 3.4. Comparison of Results between Multiplex RT-qPCR and Virus Neutralization Test Using 232 Nasal Swab Samples and Sera from 63 Clinically Healthy Calves

The results by multiplex RT-qPCR and the results by VNT against each virus, using 232 nasal swab samples and sera from 63 clinically healthy calves, are summarized in Table 3. The number of multiplex RT-qPCR-positive samples (shown Ct value ≤ 40) were BVDVs (0), BCoV (30), BToV (5), BAdV (9), BRSV (5), BPIV3 (2), and BHV1 (0). Moreover, the number of samples with significant increases in antibody titers against each pathogen were BVDV1 (28), BVDV2 (24), BCoV (28), BToV (29), BAdV (38), BRSV (21), BPIV3 (38), and BHV1 (7). These results between multiplex RT-qPCR positive and VNT were compared in BVDV1, BVDV2, BCoV, BToV, BAdV, BRSV, BPIV3, and BHV1 according to the flowchart (Figure 1 and Table 4).

The number of samples with multiplex RT-qPCR-positive, significant increase in antibody titers, and vaccination were BVDVs (0), BCoV (0), BToV (0), BAdVs (1), BRSV (2), BPIV3 (0), and BHV1 (0), respectively. The number of samples with multiplex RT-qPCR- positive, significant increase in antibody titers, and no vaccination were BVDVs (0), BCoV (16), BToV (2), BAdVs (0), BRSV (2), BPIV3 (0), and BHV1 (0), respectively. The number of samples with multiplex RT-qPCR-positive, no significant increase in antibody titers, and antibody possession were BVDVs (0), BCoV (3), BToV (1), BAdVs (3), BRSV (0), BPIV3 (0), and BHV1 (0), respectively. The number of samples with multiplex RT-qPCR-positive, no significant increase in antibody titers, and antibody no possession were BVDVs (0), BCoV (0), BToV (0), BAdVs (1), BRSV (0), BPIV3 (0), and BHV1 (0), respectively. The number of samples with multiplex RT-qPCR positive and unknown significant increase in antibody titers (No collection of pre- or post-serum) were BVDVs (0), BCoV (11), BToV (2), BAdVs (4), BRSV (1), BPIV3 (2), and BHV1 (0), respectively.

### 3.5. Comparison of Pathogen Detection between Calves with Respiratory Symptoms and Clinically Healthy Calves Using Multiplex RT-qPCR

**Figure 2:**
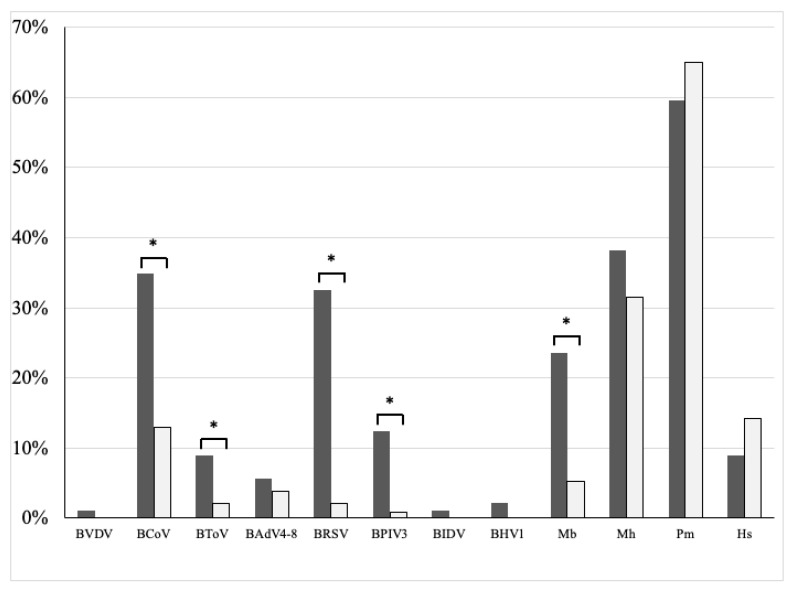
Comparison of PCR-specific gene detection rates for 12 pathogens by multiplex real-time RT-PCR between 89 nasal swabs from 89 calves with respiratory disease and 232 nasal swabs from 63 clinically healthy calves collected in Iwate prefecture during 2019 to 2021. The black bars indicate the PCR-specific gene detection rate for 12 pathogens from calves with respiratory symptoms, and white bars indicate the PCR-specific gene detection rate for 12 pathogens from clinically healthy calves. The asterisk (*) means significant differences (*p* < 0.05) between two groups by statistical analysis using Fisher’s exact test.

A total of 204 PCR-specific genes from 12 pathogens were detected using 89 nasal swab samples from 89 calves with respiratory symptoms collected in Iwate prefecture from 2019 to 2021 as follows: Pm (53/89, 59.6%), Mh (34/89, 38.2%), BCoV (31/89, 34.8%), BRSV (29/89, 32.6%), Mb (21/89, 23.6%), BPIV3 (11/89, 12.4%), BToV (8/89, 9%), Hs (8/89, 9%), BAdVs (5/89, 5.6%), BHV1 (2/89, 2.2%), BVDVs (1/89, 1.1%), and BIDV (1/89, 1.1%) (Figure 2). In contrast, the pathogen-specific genes detected by multiplex RT-qPCR using nasal swab samples from 63 clinically healthy calves are shown in Figure 2 and Table 1. The detection rates of PCR-specific genes for 12 pathogens between calves with respiratory symptoms and clinically healthy calves were compared and analyzed using Fisher’s exact test [32]. As a result, there were significant differences (*p* < 0.05) in the detection rates of BCoV, BToV, BRSV, BPIV3, and Mb between the two groups. In contrast, there were no significant differences in the detection rates of BVDVs, BAdVs, BIDV, BHV1, Mh, Pm, and Hs (Figure 2).

In the analysis by multiplex RT-qPCR using 89 calves with respiratory symptoms, 58 samples (65.2%) simultaneously detected two to five pathogens (Supplementary Table S3). In contrast, 102 (44.0%) of 232 samples from 63 clinically healthy calves simultaneously detected two or more pathogens (Supplementary Table S4).

### 3.6. Association of Pathogen Detection by Multiplex RT-qPCR Results with Significant Increase in Antibody Titers before and after Onset of the Disease in 16 Calves with Respiratoty Diseases among 63 Clinically Healthy Calves

In this study, seven calves (B1–B3, D4–D6, and G6) showed respiratory disease and nine calves (B7–B9, D7–D9, and F4–F6) were suspected to have respiratory disease among 63 clinically healthy calves during the investigation period. The multiplex RT-qPCR results from before to after the onset of the disease and the significant increase in antibody titers after the onset of the disease in these 16 calves is summarized in Table 5. The multiplex RT-qPCR results before the onset of disease in ten calves were BAdV (1), Mh (1), Pm (5), Mh and Pm (2), and undetected (1). The multiplex RT-qPCR results at the onset of the disease in eight calves were BCoV, BAdV, Mh, and Pm (1), BCoV, BRSV, Mh, and Pm (1), BCoV, BRSV, and Pm (1), BCoV, Mh, and Pm (1), BCoV and Pm (2), and BRSV and Pm (2). The multiplex RT-qPCR results, after the onset of the disease in 11 calves, were BCoV, BRSV, and Pm (1), BCoV, Mh, and Pm (4), BCoV and Pm (2), Mh and Pm (2), Mh (1), and Pm (1). A significant increase in BCoV antibodies was observed in all 12 calves in which BCoV was detected at the time of onset or after onset. A significant increase in BRSV antibodies was observed in four of five calves in which BRSV was detected at the time of onset or after onset.

## 4. Discussion

Viral and bacterial pathogens play an important role in BRD onset. Therefore, early detection of these pathogens can reduce the damage caused by BRD [33,34]. However, there are numerous pathogens associated with BRD. Therefore, an efficient method to rapidly detect them is needed. For this reason, we used multiplex RT-qPCR to simultaneously detect eight viruses and four bacteria related to BRD [27]. However, it is unclear whether the calf is actually infected with those pathogens in the test by only multiplex RT-qPCR. Hence, we confirmed a history of infection by checking significant increases in antibody titers with VNT. In this study, multiplex RT-qPCR and VNT were performed using nasal swabs or serum samples collected from 63 clinically healthy calves from seven farms and 89 BRD-infected calves from 28 farms in Iwate Prefecture from 2019 to 2021. We attempted to detect BRD-associated pathogens dominant in this region and to understand the roles of the BRD-associated pathogens in clinically healthy calves via these analyses.

To analyze the presence of BRD-associated pathogens in nasal swabs of clinically healthy calves and their relationship to real infection, we compared results between multiplex RT-qPCR and VNT against BVDV1, BVDV2, BCoV, BToV, BAdV, BRSV, BPIV3, and BHV1 using 232 nasal swab samples and 232 serum samples from 69 clinically healthy calves. Herein, we focused on the percentage of samples with a significant increase in antibody titers against BCoV, BToV, BRSV, and BPIV3 that were positive with multiplex RT-qPCR. Except for cases that were unknown by VNT, the pathogens that showed a higher percentage of VNT were BCoV (16 samples with multiplex RT-qPCR positives and significant increase in antibody titers/30 samples with multiplex RT-qPCR positives and 11 samples unknown, therefore 19 samples were adapted: 84.2%), BToV (2/3, 66.7%), and BRSV (2/2, 100%). For BPIV3, there were no applicable samples. Therefore, this suggests that the positive results by multiplex RT-qPCR could be an indicator of real infection. In addition, these findings revealed that several calves with antibody titers of 4 ≤ were shown to be positive by multiplex RT-qPCR. We speculate that those individuals infected more viruses than range that can protect them from BCoV infections by their VN titers. In addition, the positive results might be also influenced by host health condition and co-infection by several pathogens other than the amount of virus exposed.

We compared 12 BRD-related pathogens detected by multiplex RT-qPCR using 89 and 232 nasal swab samples collected from 89 calves with respiratory symptoms and 63 clinically healthy calves during 2019–2021, respectively, owing to finding pathogens that are indicators of BRD onset. As results, we revealed that BCoV, BToV, BRSV, BPIV3, and Mb have been more frequently detected in calves with respiratory symptoms compared to those detected in clinically healthy calves. In previous studies, BRSV, BPIV3, and Mb have been reported as major pathogens of BRD [13,35]. There were several previous reports that BCoV is associated with BRD as well as diarrhea [36,37]. Recently, BToV has also been suggested to be causative virus of BRD [8,9]. Taken together, our data strongly support the previous reports that these pathogens have been closely related to BRD among calves.

Pm and Mh have been reported to be resident bacteria in the nasal cavity [38]. In addition, they are also considered one of the secondary exacerbation factors of BRD. Moreover, it is known that Pm and Mh were further infected in nasal mucosa when damaged by stress and/or viruses, and therefore, symptoms of the infected animals became more severe [39,40]. Our analysis exhibited that there were no significant differences in the detection of Mh- and Pm-specific genes between BRD-infected and healthy calves. Moreover, our data also showed that Mh- and Pm-specific genes were detected several times from the same individuals. Therefore, these findings suggest that Mh and Pm might reside in the nasal cavity of calves and potential exacerbation factors. However, we did not analyze and compare cultivation and quantification of the bacteria between BRD-infected and clinically heathy calves in this study. Thus, we will attempt to perform a further analysis in the future to certify this possibility.

Our analysis found one farm (No.A) where Mh and Pm were never detected for three years. In addition, our data showed that few viruses associated with BRD were detected at the farm. The farm had a small number of cows with little introduction of cattle and no other dairy farms in the surrounding area. These facts suggest that reduction of stress by cattle movement and herd organization, and enhancement of biosecurity in cattle management might be effective for the prevention of BRD [41,42,43].

In this study, we confirmed that seven calves showed BRD symptoms (two calves of them died) out of 63 clinically healthy calves throughout the interview with farmers and diagnosis by veterinarians with experience and knowledge. In addition, there were epidemic cases of respiratory disease on three farms (B, D, and F) during the investigation period, and moreover nine calves (B7–B9, D7–D9, and F4–F6) were suspected to have the disease. Multiplex RT-qPCR and VNT were performed using samples from these 16 calves collected before and after BRD onset. Our analysis revealed one or two pathogens (mainly Mh and Pm) before BRD onset. Subsequently, mixed infections with two to four pathogens of viruses (mainly BCoV and BRSV) and bacteria (mainly Mh and Pm) were detected at BRD onset. Moreover, the number of pathogens detected using a nasal swab sample from healthy and BRD-infected calves was 1.4 and 2.3 pathogens, respectively. The pathogenesis mechanism of BRD has been suggested to be based on stress, immunosuppression, and potentially bacterial pathogens in the nasal cavity. At BRD onset, there is a shift in this homeostatic balance in the upper respiratory tract, resulting in colonization in the lower respiratory tract [12]. Moreover, the virus has been found to play a variety of roles as a stressor, including involvement in immunosuppression, invasion of pathogens into the upper or lower airways, and promotion of secondary infection by the virus [34,44,45]. Our findings might support the pathogenesis mechanism of BRD reported in previous study.

Identifying causative pathogens of BRD requires rapid testing for multiple pathogens, including viruses and bacteria. Our investigation using samples from 16 BRD-infected calves showed that at least BCoV was associated with respiratory symptoms in 12 calves (B7–B9, D4–D9, and F4–F6) on three farms (B, D, and F). Of these, six calves (D4-D9) from farm D were negative for BCoV antibodies before BRD onset and then were infected with BCoV at BRD onset.

In conclusion, we offer the application of multiplex RT-qPCR as a tool to monitor BRD causative pathogens and to detect the risk before BRD is spread within the herds. Analyses by multiplex RT-qPCR using nasal swab samples from calves with respiratory symptoms and clinically healthy calves suggests that BCoV, BToV, BRSV, BPIV3, and Mb are the main causative pathogens of BRD. Moreover, our data revealed that positive results by multiplex RT-qPCR are closely correlated, with significant increases in antibody titers by VNT regarding BCoV, BToV, and BRSV. Therefore, these facts support the idea that continuous monitoring of BRD-associated pathogens in healthy calves using multiplex RT-qPCR would contribute to early detection of BRD. Furthermore, this study found, in one of mechanism of BRD, that Mh and Pm existed in the nasal cavity before BRD onset, and that multiple pathogens of viruses and bacteria, such as BCoV, BRSV, Mh, and Pm were infected there. Therefore, we hope this methodology will be widely spread and accumulate more data in the future.

## Figures and Tables

**Table 1:** Summary of Ct values of bovine respiratory disease-associated 12 pathogens using 232 nasal swabs from 63 clinically healthy calves by multiplex real-time RT-PCR.

Farm	Carf	BVDV				BCoV				BToV				BAdV4-8				BRSV				BPIV3				BHV1				BIDV				Mb				Mh				Pm				Hs			
		1st	2nd	3rd	4th	1st	2nd	3rd	4th	1st	2nd	3rd	4th	1st	2nd	3rd	4th	1st	2nd	3rd	4th	1st	2nd	3rd	4th	1st	2nd	3rd	4th	1st	2nd	3rd	4th	1st	2nd	3rd	4th	1st	2nd	3rd	4th	1st	2nd	3rd	4th	1st	2nd	3rd	4th
A	A1	-	-	-	-	-	-	-	-	-	-	-	-	-	-	-	-	-	-	-	-	-	-	-	-	-	-	-	-	-	-	-	-	-	-	-	-	-	-	-	-	-	-	-	-	-	-	-	-
	A2	-	-	-	-	-	-	-	-	-	-	-	-	-	-	-	-	-	-	-	-	-	-	-	-	-	-	-	-	-	-	-	-	-	-	-	-	-	-	-	-	-	-	-	-	35.6	31.5	-	-
	A3	-	-	-	-	-	-	-	-	-	-	-	-	-	-	-	-	-	-	-	-	-	-	-	-	-	-	-	-	-	-	-	-	-	-	-	-	-	-	-	-	-	-	-	-	-	-	-	-
	A4	-	-	-	-	-	-	-	-	-	-	-	-	-	-	-	-	-	-	-	-	-	-	-	-	-	-	-	-	-	-	-	-	-	-	-	-	-	-	-	-	-	-	-	-	-	-	-	-
	A5	-	-	-	-	-	-	-	-	-	-	-	-	-	-	-	-	-	-	-	-	-	-	-	-	-	-	-	-	-	-	-	-	-	-	-	-	-	-	-	-	-	-	-	-	-	-	-	-
	A6	-	-	-	-	-	-	-	-	-	-	-	-	-	-	-	-	-	-	-	-	-	-	-	-	-	-	-	-	-	-	-	-	-	-	-	-	-	-	-	-	-	-	-	-	-	-	-	-
	A7	-	NT	-	-	-	NT	-	-	-	NT	-	-	37.7	NT	-	-	-	NT	-	-	-	NT	-	-	-	NT	-	-	-	NT	-	-	-	NT	-	-	-	NT	-	-	-	NT	-	-	-	NT	-	-
	A8	-	NT	-	-	-	NT	-	-	-	NT	-	-	-	NT	-	-	-	NT	-	-	-	NT	-	-	-	NT	-	-	-	NT	-	-	-	NT	-	-	-	NT	-	-	-	NT	-	-	-	NT	-	-
	A9	-	NT	-	-	-	NT	-	-	-	NT	-	-	-	NT	-	-	-	NT	-	-	-	NT	-	-	-	NT	-	-	-	NT	-	-	-	NT	-	-	-	NT	-	-	-	NT	-	-	-	NT	-	-
B	B1	-	NT	NT	NT	-	NT	NT	NT	-	NT	NT	NT	-	NT	NT	NT	-	NT	NT	NT	-	NT	NT	NT	-	NT	NT	NT	-	NT	NT	NT	-	NT	NT	NT	-	NT	NT	NT	-	NT	NT	NT	-	NT	NT	NT
	B2	-	-	-	-	-	-	-	-	-	-	-	-	36.5	-	-	-	-	25.6	-	-	-	-	-	-	-	-	-	-	-	-	-	-	-	-	-	-	-	-	35.8	-	-	32.3	-	-	-	-	-	-
	B3	-	-	-	-	-	-	-	-	-	-	-	-	-	-	-	-	-	26.3	-	-	-	-	-	-	-	-	-	-	-	-	-	-	-	-	-	-	-	-	-	37.9	37.6	30.3	29.9	30.2	-	-	-	-
	B4	-	-	-	-	-	-	-	-	-	-	-	-	-	-	-	-	-	-	-	-	-	-	-	-	-	-	-	-	-	-	-	-	-	-	-	-	-	-	-	-	-	-	-	32.8	-	-	-	-
	B5	-	-	-	-	-	-	-	-	-	-	-	-	-	-	-	-	-	-	-	-	-	-	-	-	-	-	-	-	-	-	-	-	-	-	-	-	-	-	-	36.0	29.7	-	33.9	-	-	-	-	-
	B6	-	-	-	-	-	-	-	-	-	-	-	-	-	-	37.1	-	-	-	-	-	-	-	-	-	-	-	-	-	-	-	-	-	-	-	-	-	-	-	36.7	32.9	26.3	25.4	26.4	29.6	-	-	-	-
	B7	-	NT	-	-	35.0	NT	-	37.4	-	NT	35.5	-	-	NT	-	-	-	NT	-	-	-	NT	-	-	-	NT	-	-	-	NT	-	-	-	NT	-	-	-	NT	-	-	31.3	NT	27.4	-	-	NT	-	-
	B8	-	NT	-	-	38.9	NT	-	-	-	NT	33.6	-	-	NT	-	-	29.5	NT	-	-	-	NT	-	-	-	NT	-	-	-	NT	-	-	-	NT	-	-	-	NT	-	36.7	26.0	NT	18.9	-	-	NT	-	-
	B9	-	NT	-	-	29.4	NT	-	33.6	-	NT	-	-	-	NT	-	-	-	NT	-	-	-	NT	-	-	-	NT	-	-	-	NT	-	-	-	NT	-	-	-	NT	-	37.8	29.0	NT	27.3	31.7	-	NT	-	-
C	C1	-	-	-	-	-	-	-	-	-	-	-	-	-	-	-	-	-	-	-	-	-	-	-	-	-	-	-	-	-	-	-	-	-	-	-	-	-	-	-	-	-	29.5	-	35.7	-	-	-	-
	C2	-	-	-	-	-	-	-	-	-	-	-	-	-	-	-	-	-	-	-	-	-	-	-	-	-	-	-	-	-	-	-	-	-	-	-	-	-	-	-	-	34.3	29.4	30.2	-	-	-	-	-
	C3	-	-	-	-	-	-	-	-	-	-	-	-	-	-	-	-	-	-	-	-	-	-	-	-	-	-	-	-	-	-	-	-	-	-	-	-	-	-	-	-	-	28.2	25.8	28.0	-	-	-	-
	C4	-	-	-	-	-	-	24.1	-	-	-	-	-	-	-	-	-	-	-	-	-	-	-	-	-	-	-	-	-	-	-	-	-	-	-	-	-	-	-	-	-	27.7	23.8	27.6	25.1	31.4	-	-	-
	C5	-	-	-	-	-	-	-	-	-	-	-	-	-	-	-	-	-	-	-	-	-	-	-	-	-	-	-	-	-	-	-	-	-	-	-	-	-	34.8	27.7	31.7	24.5	23.4	28.5	24.2	29.6	-	-	-
	C6	-	-	-	-	-	-	-	-	-	-	-	-	-	-	-	-	-	-	-	-	-	-	-	-	-	-	-	-	-	-	-	-	-	-	-	-	-	28.4	29.5	31.8	27.4	31.4	24.3	23.9	29.3	-	39.1	-
	C7	NT	NT	-	-	NT	NT	-	-	NT	NT	-	-	NT	NT	-	-	NT	NT	-	-	NT	NT	-	33.3	NT	NT	-	-	NT	NT	-	-	NT	NT	-	-	NT	NT	-	-	NT	NT	22.9	24.3	NT	NT	36.8	-
	C8	NT	NT	-	-	NT	NT	-	-	NT	NT	-	-	NT	NT	-	-	NT	NT	-	-	NT	NT	-	-	NT	NT	-	-	NT	NT	-	-	NT	NT	-	-	NT	NT	31.2	-	NT	NT	24.4	27.7	NT	NT	30.6	-
	C9	NT	NT	-	-	NT	NT	-	-	NT	NT	-	-	NT	NT	-	-	NT	NT	-	-	NT	NT	-	-	NT	NT	-	-	NT	NT	-	-	NT	NT	-	-	NT	NT	27.7	35.4	NT	NT	22.0	28.4	NT	NT	-	-
D	D1	-	-	-	-	-	-	-	-	-	-	-	-	-	33.0	-	-	-	-	-	-	-	-	-	-	-	-	-	-	-	-	-	-	-	-	-	-	-	29.7	30.9	-	37.6	26.7	28.6	36.7	-	-	-	-
	D2	-	-	-	-	-	-	-	-	-	-	-	-	-	-	-	-	-	-	-	-	-	-	-	-	-	-	-	-	-	-	-	-	-	-	-	-	-	-	-	-	-	-	36.0	35.5	-	-	-	-
	D3	-	-	-	-	-	-	-	-	-	-	-	-	-	-	-	-	-	-	-	-	-	-	-	-	-	-	-	-	-	-	-	-	-	-	-	-	32.1	-	37.3	-	31.3	29.1	29.1	37.0	-	-	-	-
	D4	-	-	-	-	-	-	-	33.4	-	-	-	-	-	-	-	38.6	-	-	-	-	-	-	-	-	-	-	-	-	-	-	-	-	-	-	-	-	-	-	-	35.3	25.1	26.4	27.4	32.0	-	-	-	-
	D5	-	-	-	-	-	-	-	33.5	-	-	-	-	-	-	-	-	-	-	-	-	-	-	-	-	-	-	-	-	-	-	-	-	-	-	-	-	34.6	37.8	-	-	27.3	26.0	29.0	30.9	-	-	-	-
	D6	-	-	-	-	-	-	-	26.6	-	-	-	-	-	-	-	-	-	-	-	-	-	-	-	-	-	-	-	-	-	-	-	-	35.9	-	-	-	35.6	37.2	34.0	35.3	34.0	34.2	-	23.6	-	-	-	-
	D7	-	-	-	-	-	37.3	-	-	-	-	-	-	-	-	-	-	-	-	-	-	-	-	-	-	-	-	-	-	-	-	-	-	-	-	-	36.8	-	37.6	37.4	-	29.4	31.1	23.1	27.0	-	-	-	-
	D8	-	-	-	-	-	33.0	-	-	-	-	-	-	-	-	-	-	-	-	-	-	-	-	-	-	-	-	-	-	-	-	-	-	-	-	36.8	-	37.2	35.3	37.6	-	36.0	32.8	33.4	-	-	-	-	-
	D9	-	-	-	-	-	31.3	-	-	-	-	-	-	-	-	-	-	-	-	-	-	-	-	-	-	-	-	-	-	-	-	-	-	-	-	38.3	-	-	37.0	36.2	36.9	36.8	35.7	35.8	36.4	-	-	-	-
E	E1	-	-	-	-	37.7	-	-	-	-	-	-	-	-	-	-	-	-	-	-	-	-	-	-	-	-	-	-	-	-	-	-	-	-	-	-	-	30.9	-	-	-	24.9	-	-	30.4	-	36.8	-	36.2
	E2	-	-	-	-	-	-	-	-	-	-	-	-	-	-	-	-	-	-	-	-	-	-	-	-	-	-	-	-	-	-	-	-	-	-	-	-	-	28.4	-	-	-	22.9	33.8	31.5	31.9	-	-	-
	E3	-	-	-	-	-	-	-	-	-	-	-	-	-	-	-	-	-	-	-	-	-	-	-	-	-	-	-	-	-	-	-	-	-	-	-	-	-	-	-	-	-	32.1	-	-	-	-	-	-
	E4	-	-	-	-	-	-	-	-	-	-	-	-	-	-	-	-	-	-	-	-	-	-	-	-	-	-	-	-	-	-	-	-	-	-	-	-	-	-	-	-	-	-	-	27.5	26.4	-	-	-
	E5	-	-	-	-	-	-	-	-	-	-	-	-	-	36.9	-	-	-	-	-	-	-	-	-	-	-	-	-	-	-	-	-	-	-	-	-	-	28.2	-	-	-	-	-	-	30.6	28.0	-	-	-
	E6	-	-	-	-	-	-	-	-	-	-	-	-	-	36.6	-	-	-	-	-	-	-	-	-	-	-	-	-	-	-	-	-	-	-	-	-	-	27.5	-	32.4	-	-	-	-	26.7	28.4	28.5	31.7	34.0
	E7	-	NT	-	-	-	NT	-	-	-	NT	-	-	-	NT	-	-	-	NT	-	-	-	NT	-	-	-	NT	-	-	-	NT	-	-	-	NT	-	-	-	NT	31.1	-	-	NT	30.9	-	-	NT	-	-
	E8	-	NT	-	-	30.3	NT	-	-	-	NT	-	-	-	NT	-	-	-	NT	-	-	-	NT	-	-	-	NT	-	-	-	NT	-	-	-	NT	-	-	-	NT	-	-	-	NT	36.3	-	-	NT	-	-
	E9	-	NT	-	-	-	NT	-	-	-	NT	-	-	-	NT	-	-	-	NT	-	-	-	NT	32.5	-	-	NT	-	-	-	NT	-	-	-	NT	-	-	-	NT	-	-	29.4	NT	25.0	-	31.6	NT	-	-
F	F1	-	-	-	-	32.8	-	-	-	-	-	-	-	-	-	-	-	-	-	-	-	-	-	-	-	-	-	-	-	-	-	-	-	-	-	-	-	-	-	-	-	-	27.2	26.6	29.6	-	-	-	-
	F2	-	-	-	-	-	-	-	-	-	-	-	-	-	-	-	-	-	-	-	-	-	-	-	-	-	-	-	-	-	-	-	-	-	-	-	-	30.2	36.1	-	-	29.9	25.3	36.7	31.2	-	-	-	-
	F3	-	-	-	-	-	-	28.1	-	-	-	-	-	-	-	-	-	-	-	-	-	-	-	-	-	-	-	-	-	-	-	-	-	36.4	-	-	-	-	34.8	37.2	-	32.8	33.1	35.5	36.6	-	-	-	-
	F4	-	-	-	-	35.1	36.1	-	-	-	-	-	-	-	-	-	-	27.3	-	-	-	-	-	-	-	-	-	-	-	-	-	-	-	-	-	-	-	37.5	32.0	36.0	34.4	27.2	25.2	28.1	28.0	-	-	-	-
	F5	-	-	-	-	17.2	-	-	-	-	-	-	-	-	-	-	-	-	-	-	-	-	-	-	-	-	-	-	-	-	-	-	-	-	-	-	-	-	22.3	-	34.0	29.8	22.1	26.3	28.4	-	-	-	-
	F6	-	-	-	-	27.7	-	-	-	-	-	-	-	-	-	-	-	30.7	-	-	-	-	-	-	-	-	-	-	-	-	-	-	-	-	-	-	-	-	24.7	-	34.5	34.2	22.4	-	31.2	-	-	-	-
	F7	-	-	-	-	-	-	-	-	-	-	-	-	-	-	-	39.0	-	-	-	-	-	-	-	-	-	-	-	-	-	-	-	-	-	-	-	-	29.7	36.8	-	36.3	32.6	33.5	36.0	34.1	-	-	-	37.8
	F8	-	-	-	-	33.5	37.9	-	-	-	34.7	-	-	-	-	-	37.6	-	-	-	-	-	-	-	-	-	-	-	-	-	-	-	-	-	-	-	-	-	37.7	28.1	33.0	31.7	33.1	35.9	35.1	-	-	34.4	37.0
	F9	-	-	-	-	-	-	-	-	-	-	-	-	-	-	-	-	-	-	-	-	-	-	-	-	-	-	-	-	-	-	-	-	-	-	-	-	-	37.3	-	37.6	-	28.8	34.2	29.9	-	-	-	37.4
G	G1	-	-	-	-	37.5	37.8	-	-	36.2	-	-	-	-	-	-	-	-	-	-	-	-	-	-	-	-	-	-	-	-	-	-	-	-	20.2	-	-	-	23.8	-	-	-	28.5	-	34.4	-	24.9	-	-
	G2	-	-	-	-	23.1	-	-	-	-	-	-	-	-	-	-	-	-	-	-	-	-	-	-	-	-	-	-	-	-	-	-	-	-	-	-	-	-	32.7	38.4	-	28.7	-	35.1	37.0	-	-	-	-
	G3	-	-	-	-	-	30.0	-	-	-	-	-	-	-	-	-	-	-	-	-	-	-	-	-	-	-	-	-	-	-	-	-	-	-	33.2	32.2	33.4	-	28.8	37.5	-	-	26.0	32.6	33.7	-	-	-	-
	G4	-	-	-	-	33.0	-	-	-	-	-	-	-	-	-	-	-	-	-	-	-	-	-	-	-	-	-	-	-	-	-	-	-	-	-	-	-	-	22.8	-	36.6	-	35.3	-	33.5	-	-	-	36.7
	G5	-	-	-	-	-	31.4	34.6	-	-	-	-	-	-	-	-	-	-	-	-	-	-	-	-	-	-	-	-	-	-	-	-	-	-	26.8	32.3	-	34.6	-	31.6	36.5	-	21.8	27.7	31.0	-	27.2	37.3	-
	G6	-	-	NT	NT	-	-	NT	NT	31.0	-	NT	NT	-	-	NT	NT	-	-	NT	NT	-	-	NT	NT	-	-	NT	NT	-	-	NT	NT	-	-	NT	NT	-	25.9	NT	NT	36.2	23.5	NT	NT	-	-	NT	NT
	G7	-	-	-	-	-	37.6	-	-	-	-	-	-	-	-	-	-	-	-	-	-	-	-	-	-	-	-	-	-	-	-	-	-	-	-	-	-	-	-	-	38.1	33.5	26.3	35.4	28.8	-	31.5	-	37.5
	G8	-	-	-	-	-	-	-	-	-	-	-	-	-	-	-	-	-	-	-	-	-	-	-	-	-	-	-	-	-	-	-	-	-	-	-	-	-	-	-	-	32.9	30.3	37.5	32.4	-	23.6	34.4	-
	G9	-	-	-	-	-	-	-	-	-	-	-	-	-	-	-	-	-	-	-	-	-	-	-	-	-	-	-	-	-	-	-	-	-	-	-	37.5	-	-	-	-	25.7	26.9	24.0	32.2	-	27.8	36.1	38.5

Ct values indicate averages of duplicate multiplex RT-qPCR. If either duplicate sample was negative by multiplex RT-qPCR for each pathogen, a retest was performed. Moreover, a case with no Ct value by two tests was considered as a negative. “-” means below detection limit (Ct value 40<). “NT” means not tested.

**Table 2:** Summary of antibody titers by virus neutralization tests against bovine viral diarrhea viruses (BVDV1 and BVDV2), bovine coronavirus (BCoV), bovine torovirus (BToV), bovine adenovirus serotype 7 (BAdV7), bovine respiratory syncytial virus (BRSV), bovine parainfluenza virus 3 (BPIV3), and bovine herpes virus 1 (BHV1) using 232 sera from 63 clinically healthy calves.

Farm	Calf	BVDV1				BVDV2				BCoV				BToV				BAdV7				BRSV				BPIV3				BHV1			
		1st	2nd	3rd	4th	1st	2nd	3rd	4th	1st	2nd	3rd	4th	1st	2nd	3rd	4th	1st	2nd	3rd	4th	1st	2nd	3rd	4th	1st	2nd	3rd	4th	1st	2nd	3rd	4th
A	A1	-	-	-	-	-	-	-	-	16	8	2	-	1024	64	8	4	2048	256	16	4	128	32	4	2	-	-	-	-	-	-	-	-
	A2	-	-	-	-	-	-	-	-	32	2	-	-	256	32	32	16	32	4	2	-	8	8	4	2	-	-	-	-	-	-	-	-
	A3	-	-	-	-	-	-	-	-	8	2	-	-	64	32	32	32	-	-	-	-	-	-	-	**4**	-	-	-	-	-	-	-	-
	A4	-	-	-	-	-	-	-	-	2	-	-	-	32	8	4	4	2	-	-	-	2	2	2	2	-	-	-	-	-	-	-	-
	A5	-	-	-	-	-	-	-	-	32	-	2	-	128	8	**64**	8	-	-	-	-	4	4	2	2	-	-	-	-	-	-	-	-
	A6	-	-	-	-	-	-	-	-	4	4	-	-	32	32	8	4	-	-	**8**	16	2	-	-	-	-	-	-	-	-	-	-	-
	A7	-	NT	-	-	-	NT	-	-	-	NT	-	-	128	NT	64	32	256	NT	-	-	-	NT	-	-	-	NT	-	-	-	NT	-	-
	A8	-	NT	-	-	-	NT	-	-	4	NT	-	-	512	NT	256	32	-	NT	128	32	-	NT	-	-	-	NT	-	-	-	NT	-	-
	A9	-	NT	-	-	-	NT	-	-	8	NT	2	-	64	NT	64	32	-	NT	-	**8**	-	NT	-	-	-	NT	-	-	-	NT	-	-
B	B1	32	NT	NT	NT	-	NT	NT	NT	32	NT	NT	NT	64	NT	NT	NT	256	NT	NT	NT	16	NT	NT	NT	32	NT	NT	NT	-	NT	NT	NT
	B2	4	-	-	-	-	-	-	-	16	8	16	16	16	8	2	4	64	32	8	-	4	2	**16**	16	2	**8**	**32**	8	-	-	**-**	-
	B3	32	4	-	-	-	-	-	-	16	16	16	16	32	2	2	2	256	16	-	-	8	2	**128**	128	16	32	32	8	-	-	**-**	-
	B4	8	2	-	-	8	-	-	-	32	32	32	8	64	32	8	**512**	512	64	8	**1024**	32	16	16	-	32	16	4	-	-	-	-	-
	B5	4	-	-	-	-	-	-	-	32	16	8	4	64	32	4	**1024**	32	2	-	**4096**	32	16	-	-	8	4	-	-	-	-	-	-
	B6	8	-	-	-	-	-	-	-	64	32	32	16	256	128	64	**2048**	16	8	4	4	16	4	2	2	8	8	4	4	-	-	-	-
	B7	-	NT	-	-	-	NT	-	-	128	NT	64	**256**	64	NT	2048	2048	8	NT	2	2	4096	NT	512	256	4	NT	-	**16**	-	NT	-	-
	B8	-	NT	-	-	-	NT	-	-	64	NT	64	**512**	512	NT	4096	1024	16	NT	-	-	256	NT	64	64	-	NT	-	**32**	-	NT	-	-
	B9	-	NT	-	-	-	NT	-	-	128	NT	128	128	128	NT	1024	128	128	NT	-	-	128	NT	32	32	-	NT	-	**32**	-	NT	-	-
C	C1	8	**2048**	1024	1024	-	2	2	**32**	16	16	16	16	32	**512**	256	512	4096	4096	4096	4096	8	8	8	4	-	-	-	-	-	-	-	-
	C2	2	**2048**	1024	1024	-	**16**	32	8	32	16	16	16	1024	256	256	256	4096	4096	4096	4096	4	2	2	**32**	4	8	4	2	-	-	-	**-**
	C3	-	**128**	**1024**	512	-	-	**16**	32	32	4	2	2	256	128	32	32	32	**512**	**4096**	4096	16	16	8	8	32	4	2	-	-	-	-	-
	C4	256	512	1024	512	2	**8**	4	4	64	16	8	**512**	4096	2048	1024	512	1024	2048	1024	1024	128	128	64	32	8	16	16	4	-	-	-	-
	C5	512	**2048**	2048	2048	2	**16**	32	8	64	16	16	**128**	512	128	256	128	4096	4096	4096	4096	256	128	64	64	-	-	-	-	-	-	-	-
	C6	2048	4096	2048	2048	-	**16**	8	8	64	32	16	32	4096	1024	1024	1024	128	256	256	512	64	32	64	64	8	8	16	16	-	-	-	-
	C7	NT	NT	128	**1024**	NT	NT	4	4	NT	NT	32	8	NT	NT	128	32	NT	NT	128	64	NT	NT	-	-	NT	NT	-	-	NT	NT	-	-
	C8	NT	NT	32	**1024**	NT	NT	4	8	NT	NT	16	16	NT	NT	128	32	NT	NT	4096	4096	NT	NT	-	-	NT	NT	-	-	NT	NT	-	-
	C9	NT	NT	2048	512	NT	NT	32	32	NT	NT	64	32	NT	NT	256	128	NT	NT	4096	4096	NT	NT	-	-	NT	NT	-	-	NT	NT	-	-
D	D1	-	-	-	-	-	-	-	-	16	8	16	16	16	16	4	4	-	-	-	-	4	**32**	64	64	-	-	-	-	-	-	-	-
	D2	-	-	-	-	-	-	-	-	16	16	16	8	32	16	16	16	-	-	-	-	4	**32**	64	64	-	-	-	-	-	-	-	-
	D3	-	-	-	-	-	-	-	-	16	16	16	32	256	64	32	64	1024	128	32	4	4	**32**	64	64	2	-	-	-	-	-	-	-
	D4	-	-	-	-	-	-	-	-	4	2	-	**64**	128	256	64	32	32	16	4	-	2	2	2	2	2	-	-	-	-	-	-	-
	D5	-	**1024**	512	256	-	**1024**	512	256	-	-	-	**64**	64	128	256	128	-	**128**	**4096**	4096	4	4	2	2	-	**8**	8	8	-	-	-	-
	D6	-	-	-	-	-	-	-	-	-	-	-	**64**	64	32	16	32	4	4	-	-	-	-	-	-	-	-	-	-	-	-	-	-
	D7	-	-	-	-	-	-	-	-	-	**128**	64	**256**	128	256	16	**512**	128	128	8	-	-	-	-	-	-	-	-	-	-	-	-	-
	D8	-	-	-	-	-	-	-	-	-	**64**	16	**64**	256	256	128	128	-	-	-	-	-	-	-	-	-	-	-	-	-	-	-	-
	D9	-	-	-	-	-	-	-	-	-	**16**	32	**256**	64	8	**64**	128	256	32	8	-	-	-	-	-	-	-	-	-	-	-	-	-
E	E1	32	2	-	-	8	-	-	-	32	32	16	16	32	32	32	32	16	4	-	**128**	8	4	2	32	4	-	-	**32**	-	-	-	**-**
	E2	-	-	-	-	-	-	-	-	16	16	16	16	2048	2048	1024	1024	-	-	-	**64**	-	-	-	-	64	64	64	**512**	-	-	-	-
	E3	4	-	-	-	-	-	-	-	16	16	8	16	32	8	4	**16**	64	64	16	**512**	-	-	-	-	8	4	4	**64**	-	-	-	**8**
	E4	-	-	-	**16**	-	-	-	**16**	-	-	-	-	2	4	8	8	64	64	128	**1024**	-	-	**8**	16	32	32	32	**2048**	-	-	-	**-**
	E5	4	-	-	**512**	2	-	-	**64**	-	-	-	-	4	2	4	2	1024	1024	1024	**4096**	-	-	**4**	**32**	8	8	2	**64**	-	-	-	**-**
	E6	-	-	-	**64**	-	-	-	**128**	-	-	-	-	8	4	4	4	64	64	-	**512**	-	-	-	**8**	8	**32**	4	**256**	-	-	-	**4**
	E7	8	NT	-	-	4	NT	-	-	256	NT	32	**1024**	4096	NT	16	**2048**	4	NT	-	**2048**	-	NT	8	**4096**	-	NT	4	**256**	-	NT	-	2
	E8	8	NT	-	-	-	NT	-	-	128	NT	128	**512**	16	NT	16	**1024**	16	NT	-	**4096**	-	NT	32	**4096**	2	NT	-	**64**	-	NT	-	**-**
	E9	-	NT	-	-	-	NT	-	-	2048	NT	256	256	128	NT	128	**4096**	-	NT	-	**128**	-	NT	256	256	16	NT	512	512	-	NT	-	**-**
F	F1	32	**1024**	512	128	-	**8**	8	8	32	32	16	16	128	64	32	**256**	64	8	16	**64**	128	64	32	32	4	**256**	128	32	-	-	-	-
	F2	2	**512**	512	512	-	**4**	4	4	16	32	32	64	64	64	32	**256**	128	32	-	**32**	32	16	16	8	2	**32**	16	32	-	-	-	-
	F3	-	**1024**	2048	2048	-	**16**	16	4	32	32	32	32	128	128	64	**1024**	16	**4096**	4096	2048	4	4	4	4	8	16	8	16	-	2	-	-
	F4	2	-	**512**	512	-	-	**4**	8	8	16	**64**	**1024**	32	**256**	128	256	-	**256**	**2048**	2048	4	**512**	256	128	4	4	**32**	16	-	-	2	-
	F5	16	-	**4096**	4096	4	-	**8**	16	4	**64**	64	**1024**	128	256	256	256	32	**256**	**4096**	4096	8	**512**	64	32	8	8	**32**	32	-	2	**16**	8
	F6	32	2	**2048**	512	8	-	**16**	8	4	**128**	128	**1024**	64	**512**	512	128	64	**256**	**1024**	**4096**	4	**256**	128	16	8	**32**	32	16	-	-	**4**	2
	F7	-	**64**	**1024**	2048	-	**8**	16	32	64	**256**	64	32	1024	512	**2048**	4096	64	**256**	256	**4096**	16	**64**	32	16	-	**128**	64	**512**	-	2	-	-
	F8	8	**1024**	512	512	2	**8**	16	32	64	64	32	32	2048	1024	256	**4096**	128	**512**	1024	1024	16	**128**	16	16	2	**8**	8	**64**	-	**4**	-	-
	F9	2	**128**	**2048**	512	2	**8**	8	8	2048	1024	512	128	128	128	128	256	64	**512**	**4096**	2048	2	**32**	16	8	2	**32**	32	**1024**	-	-	-	-
G	G1	128	128	64	64	16	32	32	32	32	32	16	16	64	**256**	256	1024	128	**512**	128	**512**	8	4	2	2	2	**8**	2	**16**	-	-	-	-
	G2	256	256	256	512	4	4	4	4	16	16	16	16	64	**256**	128	**1024**	64	64	16	-	4	8	8	4	4	-	-	**4**	-	2	-	-
	G3	2	**256**	256	256	-	**32**	32	32	-	**32**	16	16	128	256	128	**2048**	8	**512**	512	512	8	8	8	8	8	**128**	32	**512**	-	**8**	-	**64**
	G4	2048	512	**2048**	2048	4	8	**32**	64	-	**128**	32	32	512	**2048**	512	**2048**	4096	4096	2048	4096	16	8	16	16	2	**8**	-	**32**	-	-	-	2
	G5	256	256	**2048**	512	64	8	8	8	2	**64**	64	**256**	128	256	64	**2048**	32	4	4	4	4	2	4	4	16	2	4	**1024**	-	-	-	-
	G6	256	16	NT	NT	32	4	NT	NT	128	32	NT	NT	64	**2048**	NT	NT	128	8	NT	NT	8	4	NT	NT	128	64	NT	NT	4	-	NT	NT
	G7	-	-	-	**16**	-	-	-	**16**	512	256	**2048**	64	4096	1024	**4096**	1024	128	32	16	**1024**	-	-	-	**8**	2	-	-	**128**	-	-	-	**-**
	G8	-	-	-	**256**	-	-	-	**32**	128	64	**4096**	1024	1024	1024	**4096**	512	-	**4096**	4096	4096	2	2	-	-	8	4	-	**1024**	-	-	-	2
	G9	16	-	**512**	64	16	-	**16**	16	1024	-	-	-	2048	2048	2048	512	-	**4096**	4096	4096	-	-	-	-	8	4	**128**	**2048**	-	-	2	-

Bold indicates a significant increase. The gray shaded area indicates vaccination might have influenced an increase in each antibody titer. “-” means antibody titers of <2. “NT” means not tested. “4096” means antibody titers of 4096 or greater.

**Table 3:** Comparison of results by multiplex real-time RT-PCR and a significant increase in antibody titers by virus neutralization test against bovine viral diarrhea viruses (BVDV1 and BVDV2), bovine coronavirus (BCoV), bovine torovirus (BToV), bovine adenovirus (BAdV), bovine respiratory syncytial virus (BRSV), bovine parainfluenza virus 3 (BPIV3), and bovine herpes virus 1 (BHV1) using 232 nasal swab samples and sera from 63 clinically healthy calves.

Farm	Calf	BVDV1				BVDV2				BCoV				BToV				BAdV7				BRSV				BPIV3				BHV1			
		1st	2nd	3rd	4th	1st	2nd	3rd	4th	1st	2nd	3rd	4th	1st	2nd	3rd	4th	1st	2nd	3rd	4th	1st	2nd	3rd	4th	1st	2nd	3rd	4th	1st	2nd	3rd	4th
A	A1	-	-	-	-	-	-	-	-	16	8	2	-	1024	64	8	4	2048	256	16	4	128	32	4	2	-	-	-	-	-	-	-	-
	A2	-	-	-	-	-	-	-	-	32	2	-	-	256	32	32	16	32	4	2	-	8	8	4	2	-	-	-	-	-	-	-	-
	A3	-	-	-	-	-	-	-	-	8	2	-	-	64	32	32	32	-	-	-	-	-	-	-	**4**	-	-	-	-	-	-	-	**-**
	A4	-	-	-	-	-	-	-	-	2	-	-	-	32	8	4	4	2	-	-	-	2	2	2	2	-	-	-	-	-	-	-	-
	A5	-	-	-	-	-	-	-	-	32	-	2	-	128	8	**64**	8	-	-	-	-	4	4	2	2	-	-	-	-	-	-	-	-
	A6	-	-	-	-	-	-	-	-	4	4	-	-	32	32	8	4	-	-	**8**	16	2	-	-	-	-	-	-	-	-	-	-	-
	A7	-	NT	-	-	-	NT	-	-	-	NT	-	-	128	NT	64	32	256	NT	-	-	-	NT	-	-	-	NT	-	-	-	NT	-	-
	A8	-	NT	-	-	-	NT	-	-	4	NT	-	-	512	NT	256	32	-	NT	128	32	-	NT	-	-	-	NT	-	-	-	NT	-	-
	A9	-	NT	-	-	-	NT	-	-	8	NT	2	-	64	NT	64	32	-	NT	-	8	-	NT	-	-	-	NT	-	-	-	NT	-	-
B	B1	32	NT	NT	NT	-	NT	NT	NT	32	NT	NT	NT	64	NT	NT	NT	256	NT	NT	NT	16	NT	NT	NT	32	NT	NT	NT	-	NT	NT	NT
	B2	4	-	-	-	-	-	-	-	16	8	16	16	16	8	2	4	64	32	8	-	4	2	**16**	16	2	**8**	**32**	8	-	-	**-**	-
	B3	32	4	-	-	-	-	-	-	16	16	16	16	32	2	2	2	256	16	-	-	8	2	**128**	128	16	32	32	8	-	-	-	-
	B4	8	2	-	-	8	-	-	-	32	32	32	8	64	32	8	**512**	512	64	8	**1024**	32	16	16	-	32	16	4	-	-	-	-	-
	B5	4	-	-	-	-	-	-	-	32	16	8	4	64	32	4	**1024**	32	2	-	**4096**	32	16	-	-	8	4	-	-	-	-	-	-
	B6	8	-	-	-	-	-	-	-	64	32	32	16	256	128	64	**2048**	16	8	4	4	16	4	2	2	8	8	4	4	-	-	-	-
	B7	-	NT	-	-	-	NT	-	-	128	NT	64	**256 **	64	NT	2048	2048	8	NT	2	2	4096	NT	512	256	4	NT	-	**16**	-	NT	-	-
	B8	-	NT	-	-	-	NT	-	-	64	NT	64	**512**	512	NT	4096	1024	16	NT	-	-	256	NT	64	64	-	NT	-	**32**	-	NT	-	-
	B9	-	NT	-	-	-	NT	-	-	128	NT	128	128	128	NT	1024	128	128	NT	-	-	128	NT	32	32	-	NT	-	**32**	-	NT	-	-
C	C1	8	**2048**	1024	1024	-	2	2	**32**	16	16	16	16	32	**512**	256	512	4096	4096	4096	4096	8	8	8	4	-	-	-	-	-	-	-	-
	C2	2	**2048**	1024	1024	-	**16**	32	8	32	16	16	16	1024	256	256	256	4096	4096	4096	4096	4	2	2	**32**	4	8	4	2	-	-	-	**-**
	C3	-	**128**	**1024**	512	-	-	**16**	32	32	4	2	2	256	128	32	32	32	**512**	**4096**	4096	16	16	8	8	32	4	2	-	-	-	-	-
	C4	256	512	1024	512	2	**8**	4	4	64	16	8	**512**	4096	2048	1024	512	1024	2048	1024	1024	128	128	64	32	8	16	16	4	-	-	-	-
	C5	512	**2048**	2048	2048	2	**16**	32	8	64	16	16	**128**	512	128	256	128	4096	4096	4096	4096	256	128	64	64	-	-	-	-	-	-	-	-
	C6	2048	4096	2048	2048	-	**16**	8	8	64	32	16	32	4096	1024	1024	1024	128	256	256	512	64	32	64	64	8	8	16	16	-	-	-	-
	C7	NT	NT	128	**1024**	NT	NT	4	4	NT	NT	32	8	NT	NT	128	32	NT	NT	128	64	NT	NT	-	-	NT	NT	-	-	NT	NT	-	-
	C8	NT	NT	32	**1024**	NT	NT	4	8	NT	NT	16	16	NT	NT	128	32	NT	NT	4096	4096	NT	NT	-	-	NT	NT	-	-	NT	NT	-	-
	C9	NT	NT	2048	512	NT	NT	32	32	NT	NT	64	32	NT	NT	256	128	NT	NT	4096	4096	NT	NT	-	-	NT	NT	-	-	NT	NT	-	-
D	D1	-	-	-	-	-	-	-	-	16	8	16	16	16	16	4	4	-	-	-	-	4	**32**	64	64	-	-	-	-	-	**-**	-	-
	D2	-	-	-	-	-	-	-	-	16	16	16	8	32	16	16	16	-	-	-	-	4	**32**	64	64	-	-	-	-	-	**-**	-	-
	D3	-	-	-	-	-	-	-	-	16	16	16	32	256	64	32	64	1024	128	32	4	4	**32**	64	64	2	-	-	-	-	**-**	-	-
	D4	-	-	-	-	-	-	-	-	4	2	-	**64 **	128	256	64	32	32	16	4	-	2	2	2	2	2	-	-	-	-	-	-	-
	D5	-	**1024**	512	256	-	**1024**	512	256	-	-	-	**64 **	64	128	256	128	-	**128**	**4096**	4096	4	4	2	2	-	**8**	8	8	-	-	-	-
	D6	-	-	-	-	-	-	-	-	-	-	-	**64 **	64	32	16	32	4	4	-	-	-	-	-	-	-	-	-	-	-	-	-	-
	D7	-	-	-	-	-	-	-	-	-	**128 **	64	**256**	128	256	16	**512**	128	128	8	-	-	-	-	-	-	-	-	-	-	-	-	-
	D8	-	-	-	-	-	-	-	-	-	**64 **	16	**64**	256	256	128	128	-	-	-	-	-	-	-	-	-	-	-	-	-	-	-	-
	D9	-	-	-	-	-	-	-	-	-	**16 **	32	**256**	64	8	**64**	128	256	32	8	-	-	-	-	-	-	-	-	-	-	-	-	-
E	E1	32	2	-	-	8	-	-	-	32	32	16	16	32	32	32	32	16	4	-	**128**	8	4	2	**32**	4	-	-	**32**	-	-	-	**-**
	E2	-	-	-	-	-	-	-	-	16	16	16	16	2048	2048	1024	1024	-	-	-	**64**	-	-	-	-	64	64	64	**512**	-	-	-	-
	E3	4	-	-	-	-	-	-	-	16	16	8	16	32	8	4	**16**	64	64	16	**512**	-	-	-	-	8	4	4	**64**	-	-	-	**8**
	E4	-	-	-	**16**	-	-	-	**16**	-	-	-	-	2	4	8	8	64	64	128	**1024**	-	-	**8**	16	32	32	32	**2048**	-	-	-	**-**
	E5	4	-	-	**512**	2	-	-	**64**	-	-	-	-	4	2	4	2	1024	1024	1024	**4096**	-	-	**4**	**32**	8	8	2	**64**	-	-	-	**-**
	E6	-	-	-	**64**	-	-	-	**128**	-	-	-	-	8	4	4	4	64	64	-	**512**	-	-	-	**8**	8	**32**	4	**256**	-	-	-	**4**
	E7	8	NT	-	-	4	NT	-	-	256	NT	32	**1024**	4096	NT	16	**2048**	4	NT	-	**2048**	-	NT	8	**4096**	-	NT	4	**256**	-	NT	-	2
	E8	8	NT	-	-	-	NT	-	-	128	NT	128	**512**	16	NT	16	**1024**	16	NT	-	**4096**	-	NT	32	**4096**	2	NT	-	**64**	-	NT	-	**-**
	E9	-	NT	-	-	-	NT	-	-	2048	NT	256	256	128	NT	128	**4096**	-	NT	-	**128**	-	NT	256	256	16	NT	512	512	-	NT	-	**-**
F	F1	32	**1024**	512	128	-	**8**	8	8	32	32	16	16	128	64	32	**256**	64	8	16	**64**	128	64	32	32	4	**256**	128	32	-	-	-	-
	F2	2	**512**	512	512	-	**4**	4	4	16	32	32	64	64	64	32	**256**	128	32	-	**32**	32	16	16	8	2	**32**	16	32	-	-	-	-
	F3	-	**1024**	2048	2048	-	**16**	16	4	32	32	32	32	128	128	64	**1024**	16	**4096**	4096	2048	4	4	4	4	8	16	8	16	-	2	-	-
	F4	2	-	**512**	512	-	-	**4**	8	8	16	**64**	**1024**	32	**256**	128	256	-	**256**	**2048**	2048	4	**512**	256	128	4	4	**32**	16	-	-	2	-
	F5	16	-	**4096**	4096	4	-	**8**	16	4	**64**	64	**1024**	128	256	256	256	32	**256**	**4096**	4096	8	**512**	64	32	8	8	**32**	32	-	2	**16**	8
	F6	32	2	**2048**	512	8	-	**16**	8	4	**128**	128	**1024**	64	**512**	512	128	64	**256**	**1024**	**4096**	4	**256**	128	16	8	**32**	32	16	-	-	**4**	2
	F7	-	**64**	**1024**	2048	-	**8**	16	32	64	**256**	64	32	1024	512	**2048**	4096	64	**256**	256	**4096 **	16	**64**	32	16	-	**128**	64	**512**	-	2	-	-
	F8	8	**1024**	512	512	2	**8**	16	32	64	64	32	32	2048	1024	256	**4096**	128	**512**	1024	1024	16	**128**	16	16	2	**8**	8	**64**	-	**4**	-	-
	F9	2	**128**	**2048**	512	2	**8**	8	8	2048	1024	512	128	128	128	128	256	64	**512**	**4096**	2048	2	**32**	16	8	2	**32**	32	**1024**	-	-	-	-
G	G1	128	128	64	64	16	32	32	32	32	*32 *	16	16	64	**256**	256	**1024**	128	**512**	128	**512**	8	4	2	2	2	**8**	2	**16**	-	-	-	-
	G2	256	256	256	512	4	4	4	4	16	16	16	16	64	**256**	128	**1024**	64	64	16	-	4	8	8	4	4	-	-	**4**	-	2	-	-
	G3	2	**256**	256	256	-	**32**	32	32	-	**32 **	16	16	128	256	128	**2048**	8	**512**	512	512	8	8	8	8	8	**128**	32	**512**	-	**8**	-	**64**
	G4	2048	512	**2048**	2048	4	8	**32**	64	-	**128**	32	32	512	**2048**	512	**2048**	4096	4096	2048	4096	16	8	16	16	2	**8**	-	**32**	-	-	-	2
	G5	256	256	**2048**	512	64	8	8	8	2	**64 **	64	256	128	256	64	**2048**	32	4	4	4	4	2	4	4	16	2	4	**1024**	-	-	-	-
	G6	256	16	NT	NT	32	4	NT	NT	128	32	NT	NT	64	**2048**	NT	NT	128	8	NT	NT	8	4	NT	NT	128	64	NT	NT	4	-	NT	NT
	G7	-	-	-	16	-	-	-	**16**	512	256	**2048**	64	4096	1024	**4096**	1024	128	32	16	**1024**	-	-	-	8	2	-	-	**128**	-	-	-	-
	G8	-	-	-	256	-	-	-	**32**	128	64	**4096**	1024	1024	1024	**4096**	512	-	**4096**	4096	4096	2	2	-	-	8	4	-	**1024**	-	-	-	2
	G9	16	-	**512**	64	16	-	**16**	16	1024	-	-	-	2048	2048	2048	512	-	**4096**	4096	4096	-	-	-	-	8	4	**128**	**2048**	-	-	2	-

Bold indicates a significant increase. The gray shaded area indicates vaccination might have influenced the rise in each antibody titer. “-” means antibody titers of <2. “NT” means not tested. “4096” means antibody titers of 4096 or greater. Underlines indicate PCR-positive.

**Table 4:** A summary of combinations of positive results by multiplex real-time RT-PCR and a significant increase in antibody titers by virus neutralization test against bovine viral diarrhea viruses (BVDV1 and BVDV2), bovine coronavirus (BCoV), bovine torovirus (BToV), bovine adenoviruses (BAdVs), bovine respiratory syncytial virus (BRSV), bovine parainfluenza virus 3 (BPIV3), and bovine herpes virus 1 (BHV1).

Multiplex RT-qPCR Results	Virus Neutralization Test Results	State	BVDV1	BVDV2	BCoV	BToV	BAdV 4-8 ^a^	BRSV	BPIV3	BHV1
Positive (Ct value ≦40)	Significant increase	Vaccination	0	0	0	0	1	2	0	0
		No vaccination	0	0	16	2	0	2	0	0
	No significant increase	Antibody possession	0	0	3	1	3	0	0	0
		Antibody no possession	0	0	0	0	1	0	0	0
	Unknown	No collection of pre- or post-serum	0	0	11	2	4	1	2	0

^a^ For BAdV, multiplex RT-qPCR was performed on serotypes 4–8, whereas VNT was performed on serotype 7.

**Table 5:** Summary of the status of pathogen detection by multiplex real-time RT-PCR before and after the onset of the disease and a significant increase in antibody titers after onset by virus neutralization test in 16 calves with respiratory disease among 63 clinically healthy calves.

Calf	Timing of Onset	Respiratory Symptoms	Pathogens Detected by Multiplex RT-qPCR			Significant Increase in Antibody Titers At or After Onset	Vaccination (Timing of Vaccination)
			Before Onset	At Onset	After Onset		
B1	Between 1st and 2nd	died 17 days before 2nd	-	NT	NT	NT	None
B2	2nd	fever, cough, nasal discharge	BAdV	BRSV, Pm	Mh	BRSV (3rd), BPIV3 (2nd, 3rd)	None
B3	2nd	fever, cough, nasal discharge	Pm	BRSV, Pm	Pm	BRSV (3rd)	None
B7	Before 1st	Epidemic in herd	NT	NT	BCoV, Pm	BCoV (4th), BToV (3rd), BPIV3 (4th)	None
B8	Before 1st	Epidemic in herd	NT	NT	BCoV, BRSV, Pm	BCoV (4th), BToV (3rd), BPIV3 (4th)	None
B9	Before 1st	Epidemic in herd	NT	NT	BCoV, Pm	BToV (3rd), BPIV3 (4th)	None
D4	4th	fever, cough, nasal discharge	Pm	BCoV, BAdV, Mh, Pm	NT	BCoV (4th)	None
D5	4th	fever, cough, nasal discharge	Pm	BCoV, Pm	NT	BCoV (4th)	BVDV, BAdV7, BRSV, BPIV3, BHV1 (Between 1st and 2nd)
D6	4th	fever, cough, nasal discharge	Mh	BCoV, Mh, Pm	NT	BCoV (4th)	None
D7	Between 1st and 2nd	Epidemic in herd	Pm	NT	BCoV, Mh, Pm	BCoV (2nd, 4th), BToV (4th)	None
D8	Between 1st and 2nd	Epidemic in herd	Mh, Pm	NT	BCoV, Mh, Pm	BCoV (2nd, 4th)	None
D9	Between 1st and 2nd	Epidemic in herd	Pm	NT	BCoV, Mh, Pm	BCoV (2nd, 4th), BToV (3rd)	None
F4	1st	Epidemic in herd	NT	BCoV, BRSV, Mh, Pm	BCoV, Mh, Pm	BVDV (3rd), BCoV (3rd, 4th), BToV (2nd), BAdV7 (2nd, 3rd), BRSV (2nd), BPIV3 (3rd)	BVDV, BAdV7, BRSV, BPIV3, BHV1 (Around the 2nd)
F5	1st	Epidemic in herd	NT	BCoV, Pm	Mh, Pm	BVDV (3rd), BCoV (2nd, 4th), BAdV7 (2nd, 3rd), BRSV (2nd), BPIV3 (3rd), BHV1 (3rd)	BVDV, BAdV7, BRSV, BPIV3, BHV1 (Around the 2nd)
F6	1st	Epidemic in herd	NT	BCoV, BRSV, Pm	Mh, Pm	BVDV (3rd), BCoV (2nd, 4th), BToV (2nd), BAdV7 (2nd, 3rd, 4th), BRSV (2nd), BPIV3 (2nd), BHV1 (3rd)	BVDV, BAdV7, BRSV, BPIV3, BHV1 (Around the 2nd)
G6	Between 2nd and 3rd	died 11 days after 2nd (Sporadic respiratory symptoms in herd)	Mh, Pm	NT	NT	NT	BVDV, BAdV7, BRSV, BPIV3, BHV1 (Before 1st)

## Data Availability

Not applicable.

## Supplementary Materials

The following are available online at https://www.mdpi.com/article/10.3390/v15030669/s1, Table S1: Summary of collection dates, vaccination status, and clinical symptoms from 63 clinically healthy calves, Table S2: List of primer and probe used in multiplex RT-qPCR, Table S3: Summary of pathogen detections by multiplex RT-qPCR using 89 samples from 89 calves with respiratory symptoms.
