# Supplementary material for: Multiplex RT-qPCR Application in Early Detection of Bovine Respiratory Disease in Healthy Calves
Source: Viruses. 2023 Mar 2;15(3):669. doi: 10.3390/v15030669 (PMC10057971; doi:10.3390/v15030669)
Supplement: Supplementary file 1 [file viruses-15-00669-s001.zip › viruses-2194084-supplementary.pdf]

viruses

MDPI

Table S1. Summary of collection dates, vaccination status, and clinical symptoms from 63 clinically healthy calves.

Farm	Farm type	heads	Carf	Birth date	Collection date				Vaccination (included pathogens and date)	Respiratory symptoms
					1st	2nd	3rd	4th		
A	dairy	60	A1	2019/4/27	2019/6/28	2019/8/21	2019/9/20	2019/11/14		no
			A2	2019/2/23	2019/6/28	2019/8/21	2019/9/20	2019/11/14		no
			A3	2019/1/31	2019/6/28	2019/8/21	2019/9/20	2019/11/14		no
			A4	2020/2/18	2020/6/25	2020/8/18	2020/9/24	2020/11/20		no
			A5	2020/3/21	2020/6/25	2020/8/18	2020/9/24	2020/11/20		no
			A6	2020/2/26	2020/6/25	2020/8/18	2020/9/24	2020/11/20		no
			A7	2020/12/30	2021/6/25	NT	2021/9/22	2021/11/10		no
			A8	2021/1/12	2021/6/25	NT	2021/9/22	2021/11/10		no
			A9	2021/2/1	2021/6/25	NT	2021/9/22	2021/11/10		no
B	dairy	70	B1	2019/3/28	2019/6/28	NT	NT	NT		2019/8/4: died
			B2	2019/2/23	2019/6/28	2019/8/21	2019/9/20	2019/11/12		2nd: fever, cough, nasal discharge
			B3	2019/2/27	2019/6/28	2019/8/21	2019/9/20	2019/11/12		2nd: fever, cough, nasal discharge
			B4	2020/5/2	2020/6/25	2020/8/18	2020/9/24	2020/11/20		no
			B5	2020/4/6	2020/6/25	2020/8/18	2020/9/24	2020/11/20		no
			B6	2020/2/12	2020/6/25	2020/8/18	2020/9/24	2020/11/20		no
			B7	2021/1/29	2021/6/25	NT	2021/9/22	2021/11/10		before 1st: Epidemic in herd
			B8	2021/1/27	2021/6/25	NT	2021/9/22	2021/11/10		before 1st: Epidemic in herd
			B9	2021/1/15	2021/6/25	NT	2021/9/22	2021/11/10		before 1st: Epidemic in herd
C	dairy	90	C1	2019/1/17	2019/6/18	2019/8/21	2019/9/25	2019/11/13	BVDV1, BAdV7, BRSV, BPIV3, BHV1, 2019/4	no
			C2	2019/2/21	2019/6/18	2019/8/21	2019/9/25	2019/11/13	BVDV1, BAdV7, BRSV, BPIV3, BHV1: 2019/4	no
			C3	2019/4/18	2019/6/18	2019/8/21	2019/9/25	2019/11/13	BVDV1, BAdV7, BRSV, BPIV3, BHV1, 2019/4	no
			C4	2020/1/17	2020/6/26	2020/8/18	2020/9/24	2020/11/20	BVDV1, BAdV7, BRSV, BPIV3, BHV1, 2019/4	no
			C5	2020/1/9	2020/6/26	2020/8/18	2020/9/24	2020/11/20	BVDV1, BAdV7, BRSV, BPIV3, BHV1, 2019/4	no
			C6	2020/1/12	2020/6/26	2020/8/18	2020/9/24	2020/11/20	BVDV1, BAdV7, BRSV, BPIV3, BHV1, 2019/4	no
			C7	2021/2/11	NT	NT	2021/9/22	2021/11/10	BVDV1, BAdV7, BRSV, BPIV3, BHV1, 2021/8	no
			C8	2021/3/2	NT	NT	2021/9/22	2021/11/10	BVDV1, BAdV7, BRSV, BPIV3, BHV1, 2021/8	no
			C9	2021/3/23	NT	NT	2021/9/22	2021/11/10	BVDV1, BAdV7, BRSV, BPIV3, BHV1, 2021/8	no
D	dairy	100	D1	2019/2/13	2019/6/25	2019/8/20	2019/9/17	2019/11/19		no
			D2	2019/1/8	2019/6/25	2019/8/20	2019/9/17	2019/11/19		no
			D3	2019/1/6	2019/6/25	2019/8/20	2019/9/17	2019/11/19		no
			D4	2020/2/15	2020/6/29	2020/8/19	2020/9/28	2020/11/11		4th: fever, cough, nasal discharge

			D5	2019/12/10	2020/6/29	2020/8/19	2020/9/28	2020/11/11	BVDV1, 2, BAdV7, BRSV, BPIV3, BHV1, 2019/7	4th: fever, cough, nasal discharge
			D6	2020/1/16	2020/6/29	2020/8/19	2020/9/28	2020/11/11		4th: fever, cough, nasal discharge
			D7	2020/12/5	2021/6/29	2021/8/19	2021/9/22	2021/11/16		no
			D8	2020/12/27	2021/6/29	2021/8/19	2021/9/22	2021/11/16		no
			D9	2021/2/12	2021/6/29	2021/8/19	2021/9/22	2021/11/16		no
			E1	2019/2/4	2019/6/24	2019/8/22	2019/9/20	2019/11/12	BPIV3, BHV1, 2019/10	no
			E2	2018/11/12	2019/6/24	2019/8/22	2019/9/20	2019/11/12	BPIV3, BHV1, 2019/10	no
			E3	2019/2/17	2019/6/24	2019/8/22	2019/9/20	2019/11/12	BPIV3, BHV1, 2019/10	no
			E4	2019/12/11	2020/6/26	2020/8/19	2020/9/24	2020/11/19	BVDV1, 2, BAdV7, BRSV, BPIV3, BHV1, 2020/9	Between 1st and 2nd: Epidemic in herd
E	nursery	180	E5	2019/12/4	2020/6/26	2020/8/19	2020/9/24	2020/11/19	BVDV1, 2, BAdV7, BRSV, BPIV3, BHV1, 2020/9	Between 1st and 2nd: Epidemic in herd
			E6	2019/12/2	2020/6/26	2020/8/19	2020/9/24	2020/11/19	BVDV1, 2, BAdV7, BRSV, BPIV3, BHV1, 2020/9	Between 1st and 2nd: Epidemic in herd
			E7	2021/1/9	2021/6/30	NT	2021/9/24	2021/11/10	BPIV3, BHV1, 2021/10	no
			E8	2021/1/20	2021/6/30	NT	2021/9/24	2021/11/10	BPIV3, BHV1, 2021/10	no
			E9	2020/12/18	2021/6/30	NT	2021/9/24	2021/11/10	BPIV3, BHV1, 2021/10	no
			F1	2019/2/13	2019/6/25	2019/8/20	2019/9/17	2019/11/12	BVDV1, BAdV7, BRSV, BPIV3, BHV1, 2019/6, 7	no
			F2	2019/2/8	2019/6/25	2019/8/20	2019/9/17	2019/11/12	BVDV1, BAdV7, BRSV, BPIV3, BHV1, 2019/6, 7	no
			F3	2019/1/29	2019/6/25	2019/8/20	2019/9/17	2019/11/12	BVDV1, BAdV7, BRSV, BPIV3, BHV1, 2019/6, 7	no
			F4	2020/3/16	2020/6/30	2020/8/18	2020/9/29	2020/11/19	BVDV1, BAdV7, BRSV, BPIV3, BHV1, 2020/8	1st: Epidemic in herd
F	nursery	500	F5	2020/3/28	2020/6/30	2020/8/18	2020/9/29	2020/11/19	BVDV1, BAdV7, BRSV, BPIV3, BHV1, 2020/8	1st: Epidemic in herd
			F6	2020/3/18	2020/6/30	2020/8/18	2020/9/29	2020/11/19	BVDV1, BAdV7, BRSV, BPIV3, BHV1, 2020/8	1st: Epidemic in herd
			F7	2021/3/3	2021/6/29	2021/8/19	2021/9/16	2021/11/16	BVDV1, BAdV7, BRSV, BPIV3, BHV1, 2021/7	no
			F8	2021/3/3	2021/6/29	2021/8/19	2021/9/16	2021/11/16	BVDV1, BAdV7, BRSV, BPIV3, BHV1, 2021/7	no
			F9	2021/3/7	2021/6/29	2021/8/19	2021/9/16	2021/11/16	BVDV1, BAdV7, BRSV, BPIV3, BHV1, 2021/7	no
			G1	2019/3/5	2019/6/26	2019/8/21	2019/9/25	2019/11/13	BVDV1, BAdV7, BRSV, BPIV3, BHV1: 2019/5, 7	no
			G2	2019/2/17	2019/6/26	2019/8/21	2019/9/25	2019/11/13	BVDV1, BAdV7, BRSV, BPIV3, BHV1: 2019/5, 7	no
			G3	2019/4/6	2019/6/26	2019/8/21	2019/9/25	2019/11/13	BVDV1, BAdV7, BRSV, BPIV3, BHV1: 2019/5, 7	no
			G4	2020/1/6	2020/6/25	2020/8/19	2020/9/30	2020/11/18	BVDV1, BAdV7, BRSV, BPIV3, BHV1: 2020/5, 10	no
G	nursery	600	G5	2020/2/9	2020/6/25	2020/8/19	2020/9/30	2020/11/18	BVDV1, BAdV7, BRSV, BPIV3, BHV1: 2020/5, 10	no
			G6	2020/4/8	2020/6/25	2020/8/19	NT	NT	BVDV1, BAdV7, BRSV, BPIV3, BHV1: 2020/5	2020/8/30 died
			G7	2021/2/14	2021/6/28	2021/8/11	2021/9/28	2021/11/12	BVDV1, BAdV7, BRSV, BPIV3, BHV1, 2021/10	no
			G8	2021/2/12	2021/6/28	2021/8/11	2021/9/28	2021/11/12	BVDV1, BAdV7, BRSV, BPIV3, BHV1, 2021/10	no
			G9	2021/3/14	2021/6/28	2021/8/11	2021/9/28	2021/11/12	BVDV1, BAdV7, BRSV, BPIV3, BHV1, 2021/9	no

Table S2. List of primer and probe used in multiplex RT-qPCR.

Unit	Target pathogen	Target gene		Primer/Probe sequence 5'-3'	Reporter / quencher
1	Bovine viral diarrhea virus 1 and 2	5′ UTR	F	GRAGTCGTCARTGGTTCGAC	FAM / QSY
			R	TCAACTCCATGTGCCATGTAC	
			P	TGCYAYGTGGACGAGGGCATGC	
	Bovine coronavirus	Nucleocapsid	F	GGACCCAAGTAGCGATGAG	VIC / QSY
			R	GACCTTCCTGAGCCTTCAATA	
			P	ATTCCGACTAGGTTTCCGCCTGG	
	Bovine torovirus	Nucleocapsid	F	CGTATTCAAAACCAAAGACGTG	ABY / QSY
			R	GTGCAGTCTCATTTGCCATC	
			P	CCAGCAGTCACTATCTTTGCCATTTGA	
	Bovine adenovirus(4-8)	Hexon	F	CRAGGGAATAYYTGTCTGAAAATC	JUN / QSY
			R	AAGGATCTCTAAATTTYTCTCCAAGA	
			P	TTCATCWCTGCCACWCAAAGCTTTTTT	
2	Bovine respiratory syncytial virus	Nucleocapsid	F	GCAATGCTGCAGGACTAGGTATAAT	FAM / QSY
			R	ACACTGTAATTGATGACCCCATTCT	
			P	ACCAAGACTTGTATGATGCTGCCAAAGCA	
	Bovine parainfluenza virus 3	matrix protein	F	TGTCTTCCACTAGATAGAGGGATAAAATT	VIC / NFQ-MGB
			R	GCAATGATAACAATGCCATGGA	
			P	ACAGCAATTGGATCAATAA	
	Bovine influenza D virus	PB1	F	CAGCTGCGATGTCTGTCATAAG	ABY / QSY
			R	ACAAATTCGCAGGGCCATTA	
			P	AATGGACTTTCTCCTGGGACTGCT	
	Bovine herpesvirus 1	gE	F	CAATAACAGCGTAGACCTGGTC	JUN / QSY
			R	GCTGTAGTCCCAAGCTTCCAC	
			P	TGCGGCCTCCGGGCTTTACGTCT	
3	*Mycoplasma bovis*	*opp*D	F	TCAAGGAACCCCACCAGAT	FAM / QSY
			R	AGGCAAAGTCATTTCTAGGTGCAA	
			P	TGGCAAACTTACCTATCGGTGACCCT	
	Mannheimia haemolytica	*Lkt*D	F	CTGCAACAAAGCCGATATCTT	VIC / QSY
			R	TACGACTGCTGAAACCTTGAT	
			P	ACACATCGTCTTCCGGCACAATGA	
	Pasteurella multocida	Pm1231	F	ATCCCTGCGTTACAGAGTTTAG	ABY / QSY
			R	GACGYGGGYAGTACCATAAA	
			P	TTGATGCCTTCTTTGCGGGTTTCG	
	Histophilus somni	31kD	F	GCAATGATGTACCWGCCAAAG	JUN / QSY
			R	CCTTCAGCTCACCATTACCATA	
			P	TTGCTTACGTCCAAACCGTCGTGT	

Table S3. Summary of pathogen detections by multiplex RT-qPCR using 89 samples from 89 calves with respiratory symptoms.

Number of samples	Pathogens	BVDV	BCoV	BToV	BAdV 4-8	BRSV	BPIV3	BHV1	BIDV	Mb	Mh	Pm	Hs
1			+	+	+						+	+	
3			+			+	+			+	+		
1			+			+				+	+	+	
1	5		+				+			+	+	+	
1			+				+			+	+		+
1			+							+	+	+	+
2						+				+	+	+	+
2			+			+				+	+		
1			+			+	+				+		
1	4		+				+			+	+		
1			+							+	+	+	
1				+		+					+	+	
1										+	+	+	+
1		+	+								+		
2			+	+								+	
3			+			+						+	
1			+					+					+
1				+		+						+	
1	3			+							+	+	
3					+						+	+	
5						+					+	+	
1						+		+				+	
1						+				+		+	
1										+	+	+	
1											+	+	+
2			+	+									
1			+			+							
1			+							+			
1			+								+		
4			+									+	
1	2				+							+	
2						+						+	
1							+					+	
1									+			+	
1										+		+	
1										+			+
4											+	+	
2	1		+										

5						+						
3							+					
2										+		
11												+
8	0											

Table S4. Summary of pathogen detections by multiplex RT-qPCR using 232 samples from 63 clinically healthy calves.

Number of samples	Pathogens	BVDV	BCoV	BToV	BAdV 4-8	BRSV	BPIV3	BHV1	BIDV	Mb	Mh	Pm	Hs
2	5		+							+	+	+	+
1	4		+	+							+	+	
1			+			+					+	+	
1			+		+						+	+	
1			+							+	+	+	
1			+							+		+	+
2					+						+	+	+
2	3		+			+						+	
8			+								+	+	
1			+									+	+
2					+						+	+	
4										+	+	+	
1										+		+	+
6											+	+	+
1	2		+	+									
7			+									+	
3				+								+	
1					+								+
2						+						+	
2							+					+	
3										+		+	
36											+	+	
3											+		+
11												+	+
4	1		+										
3					+								
6											+		
53												+	
5													+
59	0												
